# *Theileria annulata* Subtelomere-Encoded Variable Secreted Protein-TA05575 Binds to Bovine RBMX2

**DOI:** 10.3389/fcimb.2021.644983

**Published:** 2021-02-26

**Authors:** Zhi Li, Junlong Liu, Shuaiyang Zhao, Quanying Ma, Aihong Liu, Youquan Li, Guiquan Guan, Jianxun Luo, Hong Yin

**Affiliations:** ^1^ State Key Laboratory of Veterinary Etiological Biology, Key Laboratory of Veterinary Parasitology of Gansu Province, Lanzhou Veterinary Research Institute, Chinese Academy of Agricultural Science, Lanzhou, China; ^2^ Jiangsu Co-Innovation Center for the Prevention and Control of Important Animal Infectious Disease and Zoonose, Yangzhou University, Yangzhou, China

**Keywords:** *Theileria annulata*, TA05575, Yeast-two hybrid, Co-IP, BiFC, RBMX2-like

## Abstract

Tropical theileriosis is the disease caused by tick-transmitted apicomplexan parasite *Theileria annulata*, which has ability to transform bovine leukocytes, including B cells, macrophage cells, and dendritic cells. The *T. annulata* transformed cells are characterized as uncontrolled proliferation and shared some cancer-like phenotypes. The mechanism of the transformation by *T. annulata* is still not understood well. In previous reports, the subtelomere-encoded variable secreted proteins (SVSP) of *T. parva* were considered to contribute to phenotypic changes of the host cell, but the role of SVSP of *T. annulata* in host-pathogen relationship remains unknown. In the present study, a member of SVSP family, TA05575 of *T. annulata* was selected as the target molecule to analyze its expression profiles in different life cycle stages of *T. annulata* by qPCR and investigate its subcellular distribution of different passages of *T. annulata* transformed cells using confocal experiments. From the results, the transcription level of TA05575 at schizont stage was significantly higher than the other two life stages of *T. annulata*, and the protein of TA05575 was mainly distributed in nucleus of *T. annulata* infected cells. In addition, the potential proteins of host cells interacting with TA05575 were screened by Yeast-two hybrid system. The results of Co-IP experiment confirmed that TA05575 interacted with RBMX2-like protein that participated in transcription regulation of cells. In addition, a novel BiFC assay and flow cytometry were carried out, and the results further revealed that TA05575-RBMX2-like pair was directly interacted in cell context. Moreover, this interacting pair was found to distribute in intracellular compartments of HEK293T cells by using confocal microscopy. The results of the present study suggest that TA05575 may contribute for cells transformation due its distribution. According to the function of RBMX2, the interaction of TA05575 and RMMX2-like will provide a new information to further understand the mechanisms of cells transformation by *T. annulata*.

## Highlights

1. We firstly discovered TA05575 is mainly expressed in schizont stage of *T. annulata*.
2. We firstly observed TA05575 protein is mainly distributed in nucleus of *T. annulata-*transformed cells.
3. BiFC and Co-IP assays showed that TA05575 interacted directly with RBMX2-like protein in cell context, and subcellular colocalization of this pair is in the intracellular compartments.

## Introduction 

*Theileria annulata* is a bovine-specific pathogen that causes tropical theileriosis in tropical and subtropical countries, including Asia (India, China), Africa (Egypt, Sudan), Middle East, Europe (Portugal) (Al-Deeb et al., 2015; Anupama et al., 2015; Liu et al., 2015; Gomes et al., 2016; El-Dakhly et al., 2018; Mohammed-Ahmed et al., 2018). The tick-borne diseases for cattle constraints livestock industry, which leads to the economic loss of USD 384.3 million per annum because of the high cost of anti-tick control and treatment, bovine mortality, and decreased animal production (Rajendran and Ray, 2014; Medjkane and Weitzman, 2020). *T. annulata* can infect bovine macrophages, B cells, and dendritic cells whereas *T. parva* invades T cells and B lymphocytes of cattle, which are referred as “schizont-transforming species.” *T. annulata* schizont-infected cells was confirmed has some cancer hallmarks, including uncontrolled proliferation, metastasis, genomic instability (Hayashida et al., 2013; Cheeseman and Weitzman, 2015; Tretina et al., 2015). Many studies were focused on host cell signaling pathways affected by the molecules of *T. annulata*, including end-binding protein 1 (EB1) (Woods et al., 2013), *T. annulata* Peptidyl-Prolyl Isomerase1 (TaPin1) (Villoutreix et al., 2015; Marsolier et al., 2019), and *T. annulata* surface protein (TaSP) (Seitzer et al., 2010), yet the key proteins of *T. annulata* directly responsible for host cell transformation remain unknown.

*T. annulata* contains two large multigene families, which are SVSP (subtelomere-encoded variable secreted protein) and TashAT (*Theileria annulata* schizont AT-hook protein). Although some previous studies have proposed that SVSP family, the largest multigene family in *T. annulata*, maybe associated with host cell transformation, invasion, and immune evasion (Barry et al., 2003; Schmuckli et al., 2009; Weir et al., 2010; Tretina et al., 2015), the detailed roles of SVSP multigene family in host-parasite interactions are unclear. The atypical codon usage, diversity of length, and extensive nucleotide variability are unique characteristics in SVSP multigene family due to its subtelomere localization (Weir et al., 2010), which indicates that the members of this multigene family maybe play an important role in manipulating host cell signaling by binding to various host cell proteins.

Therefore, exploring to the host cell molecules interacting with SVSPs is absolutely necessary at present that will yield a new sight into molecular mechanism of *Theileria*-induced cancer features. In our study, we revealed that the direct interactions between one member of SVSP multigene family-TA05575 and host cell proteins.

## Material and Methods

### Cell Culture

*Theileria annulata* schizont-infected cells were preserved at the Vector and Vector borne Disease (VVBD) team, Lanzhou Veterinary Research Institute (LVRI), China. And the cells were cultured using RPMI 1640 medium (Biological Industries, Kibbutz Beit Haemek, Israel) containing 10% fetal bovine serum (Biological Industries, Kibbutz Beit Haemek, Israel) and 100 mg/ml penicillin/streptomycin (Gibco, CA, USA) in an incubator with 5% CO_2_ at 37°C. HEK 293T cells were purchased from the China Center for Type Culture Collection, Shanghai, China. The cells were maintained in DMEM (Gibco, NY, USA) plus 10% fetal bovine serum (Gibco, NY, USA).

### Quantitative Real-Time Polymerase Chain Reaction (qRT-PCR)

Total RNA of different life cycle stages of *T. annulata* was extracted by using RNeasy Mini kit (QIAGEN, Dusseldorf, Germany) based on the manufacturer’s instruction. After measurement of the concentration and quality, 1 microgram of RNA was used for cDNA synthesization by using PrimeScript™ RT reagent kit with gDNA Eraser (Perfect Real Time) (Takara, Dalian, China). The specific primers of this gene were designed based on the sequences deposit online (Piroplasma DB No: TA05575), the sequence of primers as following: TA05575-F (5′-ACCACCAAGCTCCACTCCAT-3′) and TA05575-R (5′-ACTCCTTTGCCACCAGGATCT-3′). qRT-PCR was performed with the SYBR Premix ExTaq reagents (TaKaRa, Dalian, China) and used the Agilent Technologies Stratagene MX3005P (CA, USA). The transcription level of β-actin of *T. annulata* was used for normalization. The specific primers for β-actin of *T. annulata* were β-actin-F (5′-GAGACCACCTACAACAGCATCATG-3′) and β-actin-R (5′- CACCTTGATCTTCATGGTGCTGGG-3′). Relative amounts of transcripts for TA05575 in different life cycle stages and passages of *T. annulata*-infected cells were determined by using the comparative cycle threshold (2^-ΔΔ^ *^CT^*) method.

### Immunoblotting Analysis

Cells were lysed in lysis buffer (Beyotime, Shanghai, China, #P0013B) contained with protease inhibitor (Roche, Basel, Switzerland, #4693132001) and phosphatase inhibitor cocktail (Roche, Basel, Switzerland, #4906845001) for 30 min on ice. The cell lysates were then centrifuged at 14,000 × g at 4 °C for 10 min. Following the centrifuge, the supernatants were collected, and the protein concentration was measured by using Pierce™ BCA Protein Assay Kit (Thermo Fisher Scientific, MA, USA, #23225). Equal amounts of each protein samples were electrophoresed in SDS-PAGE based on the standard protocol, and then transferred onto a PVDF membrane (Millipore, MA, USA). The PVDF membrane was subsequently blocked with 5% bovine serum albumin (BSA) (Amresco, WA, USA) in TBST (50 mM Tris-HCl, 150 mM NaCl, 0.05% Tween-20) buffer for 2 h at room temperature. Following three times washes with TBST buffer, the membrane was incubated with the appropriate primary antibodies at 4°C for overnight. After washing four times with TBST buffer, the membrane was incubated with HRP-coupled secondary antibodies for 1 h at room temperature. Finally, the membrane was washed four times with TBST buffer, and visualized using SuperSignal™ West Pico PLUS Chemiluminescent Substrate (Thermo Fisher Scientific, MA, USA, #34577). GAPDH loading control Monoclonal Antibody (GA1R) (Thermo Fisher Scientific, MA, USA, #MA5-15738) was used to normalize protein levels of HEK 293T cells.

### Subcellular Distribution of TA05575 in *T. annulata* Infected Cell Lines

The CELLO v.2.5, a subCELlular LOcation predictor (http://cello.life.nctu.edu.tw/), was used to evaluate the subcellular location of TA05575 in *T. annulata*-infected cells. The cells of different passages (F10, F20, F55, F110, and F165) were cultured onto the glass slides (NEST) in a 12-well cell culture plate at a density of 2.0–3.0 × 10^5^ cells/ml for each well for 24 h. For confocal experiment, cells were washed with PBS three times, and fixed with 4% paraformaldehyde for 30 min at room temperature. Following three times wash with PBS, the PBS with 0.5% Triton X-100 was added for permeabilizing the cells at room temperature for 15 min. After washing with PBS, the cells were incubated for blocking in PBS containing 3% BSA for 1 h at 37°C. Primary antibody against TA05575 derived from rabbit was prepared by DETABIO Inc (Nanjing, China). Five hundred μl primary antibody, which diluted was with PBS containing 1% BSA at the ratio 1:100, was added into the wells and incubated at 4°C for overnight. Following five times washing with PBS, the cells were probed with 500 μl goat anti-rabbit Alexa Fluor 488 antibody (Life Technologies, MA, USA) at a 1:1,000 dilution in PBS plus with 1% BSA at 37°C for 1 h. DNA and actin of the cells were stained with Hoechst 3342 (Life Technologies, MA, USA) and Alexa Fluro™ 594 Phalloidin (Life Technologies, MA, USA), respectively. The cells were observed by a confocal microscope (Leica TCS) with a 63× oil objective after an additional five times washes with PBS. No less than100 cells of different passages of *T. annulata*-infected cells were randomly examined per slide, and the most representative images of each glass slide were shown in the present study.

### Construction and Identification of Bait Plasmid

The target sequence of TA05575 was obtained from the cDNA of *T. annulata*-infected cells using the PCR with its specific primers. The specific primer TA05575 was designed based on the TA05575 reference sequence and the sequences are as following: TA05575-F (5′-CCGGAATTCCGGATGAATAAATGTGTAACATAT-3′) and TA05575-R (5′-TGCACTGCAGTGCATTTGTCATCTTTTCCAGAACT-3′), and the enzyme restriction sites are underlined. The obtained PCR product for TA05575 was purified by using the Cycle-Pure kit (OMEGA Bio-Tek, Doraville, USA). Then both the purified PCR products of TA05575 and pGBKT7 plasmid were digested with Thermo Scientific™ FastDigest restriction enzymes *EcoR I* (Thermo Fisher Scientific, MA, USA, #FD0274) and *Pst I* (Thermo Fisher Scientific, MA, USA, #FD0614), respectively, which was followed by ligation step. Finally, the bait plasmid (TA05575-pGBKT7) was further identified by sequencing (Sangon Biotech, Shanghai, China) and bioinformatic analysis.

### Determination of Autoactivation and Toxicity of the Recombinant Bait Plasmid

To investigate whether the TA05575-pGBKT7 bait plasmid has the abilities of autoactivation and toxicity, the pGBKT7 plasmid (empty vector control) and the bait plasmid were respectively transformed into Y2H Gold component cells using Quick & Easy Yeast Transformation Mix (Clontech, CA, USA) according to the manufacturer’s instructions. Then, the transformants were maintained on the agar plates contained different components, including SD/-Trp (SDO), SD/-Trp/X-α-Gal (SDO/X), and SD/-Trp/X-α-Gal/AbA (SDO/X/A) for 3–5 days at 30°C. Only the color of colonies shown white on SDO plates and pale on SDO/X plates, while no colonies on SD/X/A plates, the bait plasmid could be considered not to be autoactivated. If the size of bait plasmid colonies cultured on SDO and SDO/X plates is remarkably smaller than colonies of the empty vector that indicate the bait plasmid is toxic. Only in the case of the bait plasmid are neither toxicity nor autoactivation, it is possible to perform the Y2H screening for TA05575 gene.

### Screen the Interaction Proteins With SVSP449 Using Y2H System

To screen the host cell proteins that might be interact with TA05575 of *T. annulata*, the Y2H system was performed. Firstly, the bait plasmid (TA05575-pGBKT7) and prey plasmid (the cDNA library derived from bovine B cells) (Zhao et al., 2017) were co-transformed into Y2H Gold component cells at the library scale using Yeastmaker™ Yeast Transformation System 2 (Clontech, CA, USA). Then, the transformants were cultured on DDO/X/A agar plates at 30°C for 5 days. Finally, the blue colonies were selected and plated on QDO/X/A agar plates for continued culture at 30°C for 3–5 days. Moreover, the plasmids of pGADT7-T and pGBKT7-Lam were used as negative control, pGADT7-T and pGBKT7-53 plasmids served as positive controls, both negative and positive controls were co-transformed into Y2H Gold cells at the small scale according to the standard protocol, respectively. Co-transformants were plated onto the DDO and DDO/X/A agar plates at 30°C for 3–5 days, respectively. Following the Y2H screening, identification of blue colonies cultured on the QDO/X/A agar plates with PCR was carried out using Matchmaker™ Insert Check PCR Mix (Clontech, CA, USA) according to the protocol provided by the manufacture.

### Rescue and Analysis of the Putative Prey Plasmids

Based on the results of colony PCR, the potential prey plasmids were extracted from the blue colonies picked from QDO/X/A agar plates using Easy Yeast Plasmid Isolation kit (Clontech, CA, USA) according to user manual. Three μl of extracted prey plasmids were subsequently transformed into *E. coli* DH5α competent cells (TaKaRa, Dalian, China). The possible positive prey plasmid DNA was extracted from transformants using PureLink™ HiPure Plasmid Midiprep kit (Thermo Fisher Scientific, MA, USA, #K210005) and sequenced.

The sequence of putative prey plasmids was analyzed by using the BLAST function of NCBI for confirming the potential interacting genes of host cell. Meanwhile, the structure and biological function of identified prey genes of bovine were analyzed by the SMART (http://smart.embl-heidelberg.de/) and UniProt database (http://www.uniprot.org/).

### Expression and Subcellular Localization of SVSP449 and Its Potential Binding Proteins

The recombinant plasmids (p3×Flag-CMV-prey genes) were constructed by cloning prey genes into the *Hind III* (Thermo Fisher Scientific, MA, USA, #FD0504) and *BamH I* (Thermo Fisher Scientific, MA, USA, #FD0054) digested p3×Flag-CMV vector. In addition, the pcDNA3.1- TA05575-Myc recombinant plasmid was constructed by cloning the TA05575 contained a C-terminal Myc tag into the pcDNA3.1 vector that digested with *BamH I* and *Xho I* (Thermo Fisher Scientific, MA, USA, #FD0694). To observe the expression and subcellular distribution of TA05575 and its potential interacting proteins, the confocal experiment was performed. Firstly, the HEK293T cells were seeded on glass slides in six-well cell culture plate. Then, the recombinant TA05575 and its corresponding prey plasmid were transfected or co-transfected into the cells using Lipofectamine 3000 (Thermo Fisher Scientific, MA, USA, #L3000015) when the confluent rate of the cells was 70–90%. For the immunofluorescence assay, the cells were washed, fixed, permeabilized, and blocked according to the above mentioned protocol after 24 h of transfection. The cells were incubated with mouse anti-Myc tag monoclonal antibody (CST, #2276S) or rabbit anti-Flag tag polyclonal antibody (Sigma, #F7425) at 4°C overnight, respectively. Following washed with PBS, the cells were stained with goat anti-mouse Alexa Fluor 594-or donkey anti-rabbit 488-conjugated secondary antibodies- (Life Technologies, MA, USA) according to the user manual. The nuclear of cells was stained with Hoechst33342 (Life Technologies, MA, USA). The cells were examined with confocal microscope (Leica TCS) using a 63× oil objective. For each slide, more than 100 cells were randomly visualized, and the presented images we selected were the most representative in our experiment.

### Co-IP Assay

Co-IP assay was used to identify the interactions of TA05575 and the prey proteins in HEK293T cells. The cells were cultured in 10-cm cell culture dishes (Thermo Fisher Scientific, MA, USA) at an initial density of 2 × 10^6^ cells per dish. Fourteen hours later, 10 µg of TA05575 plasmid and 10 µg of the prey plasmids were co-transfected into the cells. After 48 h of transfection, the cells were washed two times with cold PBS and lysed with 600 μl of IP/lysis buffer (Thermo Fisher Scientific, MA, USA) containing the protease inhibitor (Roche, Basel, Switzerland, #4693132001) and phosphatase inhibitor cocktail (Roche, Basel, Switzerland, #4906845001) on ice. Then, the supernatants of cell lysate were obtained by centrifuging at 16,000 × g at 4°C for 10 min. The immunoprecipitation experiment was carried out with an anti-Flag tag monoclonal antibody derived from mouse (Sigma, #F1804) using a Pierce™ Co-Immunoprecipitation kit (Thermo Fisher Scientific, MA, USA, #26149). In addition, p3×Flag-CMV and pcDNA3.1 plasmid served as negative controls, were also co-transfected into HEK293T cells. The elution from the Co-IP experiment was analyzed with western blotting. Anti-Flag tag monoclonal antibody (Sigma, #F1804) and anti-Myc tag monoclonal antibody derived from rabbit (CST, #2278S) were used to identify its target protein, respectively.

### BiFC Assay

To further confirm the interactions of TA05575 with its prey proteins, the BiFC assay was used to identify their intracellular interactions. The BiFC assay depends on the link of two nonfluorescent complementary fragments of Venus, a variant of green fluorescent protein (GFP). Two Venus fragments of the BiFC vectors are fused with the bait and prey proteins, respectively. Once the expressed SVSP449 and its partner interact to each other, they are brought in proximity to each other, leading to structural complementation and stimulate a bright fluorescent signal (Kerppola, 2008). Moreover, the assay can also visualize the subcellular localization of the interacted proteins (Kerppola, 2008).

The vectors of pBiFC-VN173 (Addgene, #22010) and pBiFC-VC155(Addgene, #22011) were purchased from Addgene (Shyu et al., 2006). The TA05575 and its prey plasmids were cloned into the pBiFC-VN173 vector after double enzymes digestion with *Hind III* and *Sal I* (Thermo Fisher Scientific, MA, USA, #FD0644). The recombinant plasmids of pBiFC-VC155-TA05575 or its prey genes were created by cloning the target gene into the pBiFC-VC155 vector, which was digested with *Sal I* and *Kpn I* (Thermo Fisher Scientific, MA, USA, #FD0524) enzymes. The pBiFC-VN173- TA05575/prey recombinant plasmids, and pBiFC-VC155-TA05575/prey plasmids were transfected individually into HEK293T cells. Moreover, the pairwise of pBiFC-VN173- TA05575/pBiFC-VC155-prey and pBiFC-VN155-TA05575/pBiFC-VC173-prey plasmids were co-transfected into HEK293T cells using Lipofectamine 3000. In addition, the pBiFC-VN173 and pBiFC-VC155 empty plasmids were also transfected into HEK293T cells, which were served as the blank controls. The confocal microscopy experiments were performed to observe the fluorescent signal and subcellular localization in cell context. For confocal experiment, the cells were incubated with mouse anti-Flag tag monoclonal antibody at a dilution ratio of 1:200 or anti-HA tag monoclonal antibody produced in rabbits (CST, #3724S) at a dilution ratio of 1:800 after fixing, permeabilizing, and blocking of HEK293T cells at 4°C overnight. Followed washing the cells with PBS, donkey anti-rabbit 594- or goat anti-mouse Alexa Fluor 594-conjugated secondary antibodies (Life Technologies, MA, USA) was incubated with the cells at dilution ratio 1:1,000 for 1 h at room temperature, respectively. The cells were visualized with confocal microscope (Leica TCS) after staining cells nuclear with Hoechst 33342 (Life Technologies, MA, USA).

### Flow Cytometry Analysis

HEK293T cells were seeded at initial density of 0.5 × 10^6^ cells/ml in six-well cell culture plates (Corning, USA). The pBiFC-VN173-TA05575 and its potential plasmids, the pBiFC-VC155-TA05575 and its corresponding plasmids were transfected individually into HEK293T cells. At the same time, pBiFC-VN173-TA05575/pBiFC-VC155-prey pair and pBiFC-VN155-TA05575/pBiFC-VC173-prey pair were also co-transfected into HEK293T cells when the confluent rate of cells was 70–90%. The fluorescence signal of transfected cells was determined by fluorescence microscopy after 48 h of transfection. The cells were harvested and washed with PBS, and then applied on BD Accuri™ C6 Plus Flow Cytometer (USA) for analysis of the mean fluorescence intensity (MFI) of the cells.

### Data and Statistical Analysis

Statistical analysis was performed using GraphPad Prism 7 software. The significance of differences between samples was evaluated using unpaired two-tailed Student’s *t*-test. The variance was assessed by calculating the standard error of the mean (SEM) in each group. All experiments were carried out independently at least three times. *p < 0.05; **p < 0.01; ***p <0.001; ****p < 0.0001 and NS, not significant (p < 0.05).

## Results

### Analysis of TA05575 at mRNA Levels in Different Life Cycle Stages of *T. annulata*

**Figure 1:**
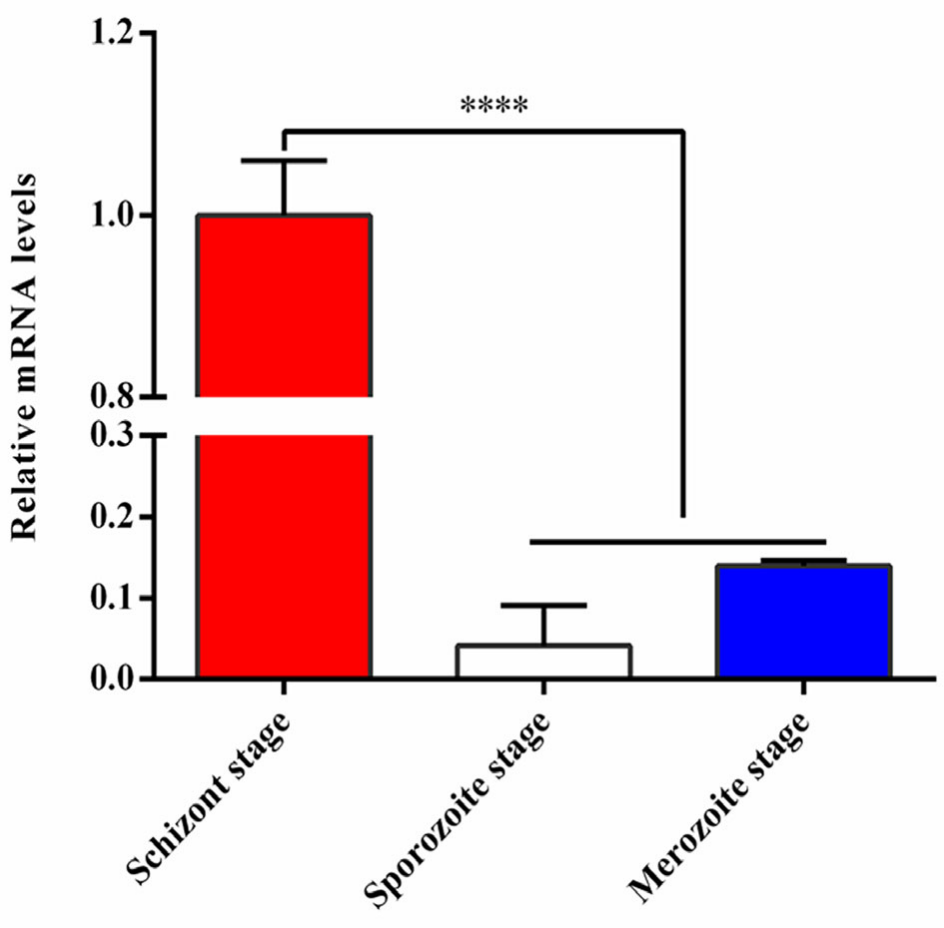
Expression analysis of TA05575 at mRNA level in different life cycle stages of *T. annulata*. ****p < 0.0001.

The relative mRNA levels of TA05575 in schizont stage of *T. annulata* was significantly higher than that of sporozoite and merozoite stages based on the results of qPCR (**Figure 1**), so it was mainly expressed in *T. annulata* schizont stage, which was consistent with the reports of previous studies (Pain et al., 2005; Shiels et al., 2006; Schmuckli et al., 2009).

### Subcellular Colocalization of TA05575 in *T. annulata-*Infected Cell Lines

**Figure 2:**
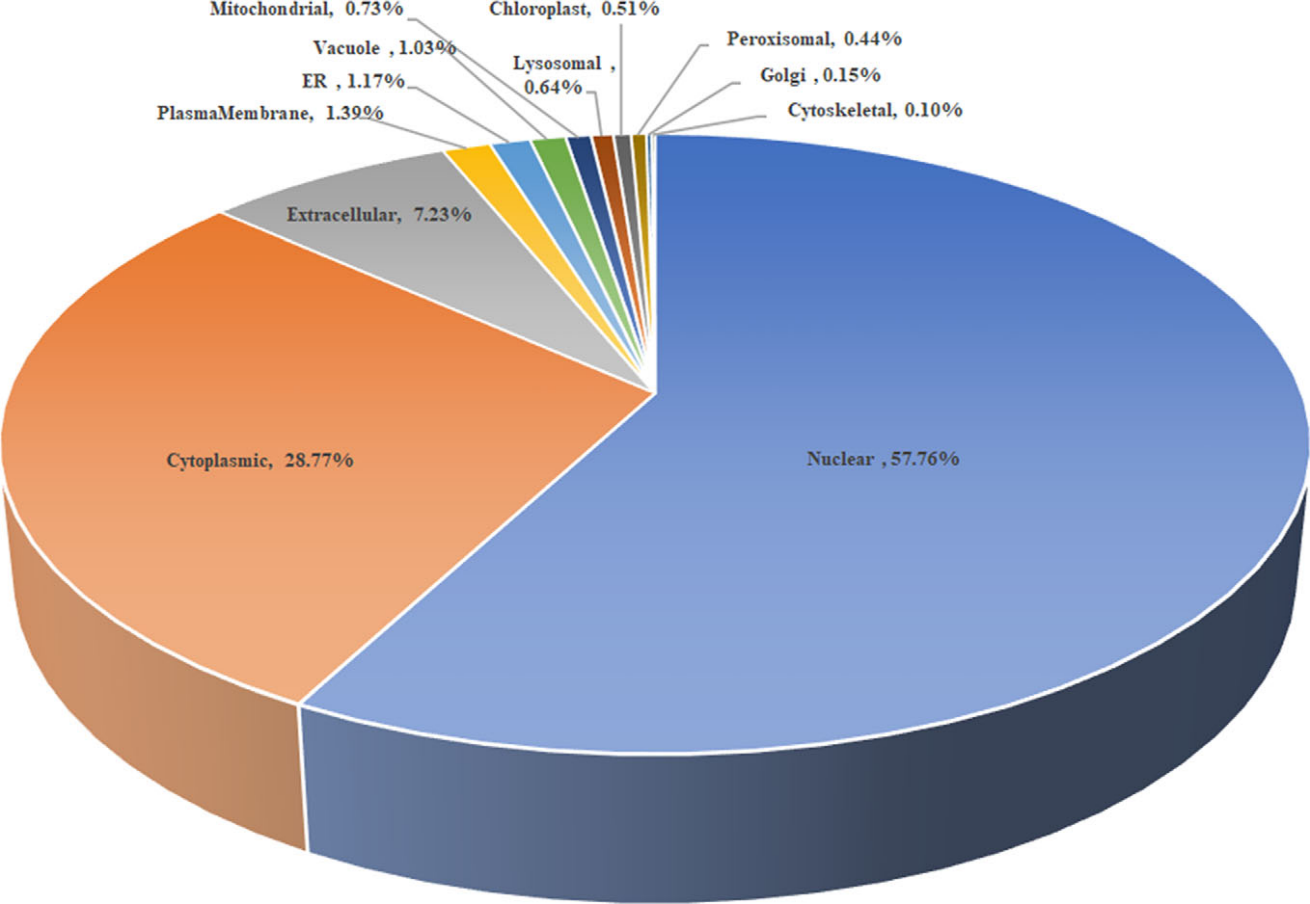
The scheme of subcellular distribution of TA05575 in *T. annulata*-infected cell using CELLO v.2.5. ER represents the endoplasmic reticulum.

**Figure 3:**
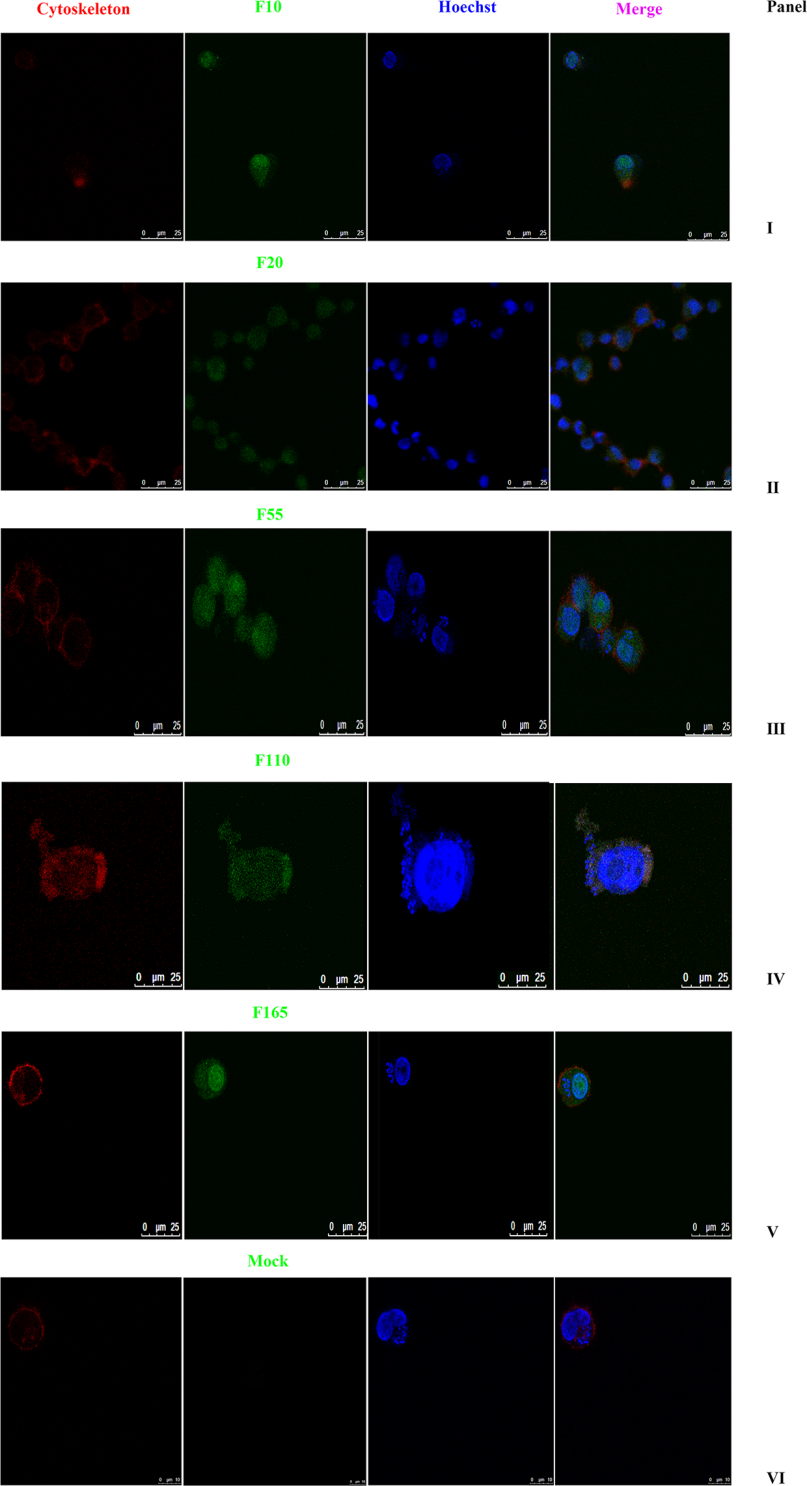
Subcellular localization of TA05575 in *T. annulata*-infected cells. Panels I to V displayed the subcellular localization of TA05575 in different culture passages (F10, F20, F55, F110, and F165) of *T. annulata-*infected cells. Panel VI was the negative control. The TA05575 (Panel I to V) were incubated with rabbit polyclonal antibody against TA05575 and then stained with an Alexa Fluor 488-conjugated goat anti-rabbit antibody. Hoechst 33342 was used to label cell nuclei, Alexa Fluor™ 594 phalloidin was used to label the cytoskeleton. Subcellular localization of TA05575 was visualized by confocal microscopy. Scale bar = 25 μm.

As shown in **Figure 2**, the subcellular distribution of TA05575 was mainly in nucleus (57.76%) and cytoplasm (28.77%) of the *T. annulata*-infected cells based on the results of CELLO v.2.5. Moreover, the findings of confocal microscopy showed that the subcellular distribution of TA05575 was mainly in nucleus and cytoplasm in different passages (F10, F20, F60, F110, and F165) of *T. annulata*-infected cells (**Figure 3**), which was in consistent with the results of CELLO v.2.5.

### Identification and Bioinformatic Analysis of TA05575

A fragment of TA05575 was successfully amplified from the cDNA of *T. annulata*. In the present study, the length of the TA05575 ORF was 1,350 bp that encoding 450 amino acids, with the predicted size of the TA05575 was about 52 kDa. The TA05575 was predicted to contain a signal peptide from 1 to 21 amino acids and a nuclear localization signal sequence (NLSs) from 217 to 234 amino acids but no GPI-anchors, based on the SignalP-5.0 (http://www.cbs.dtu.dk/services/SignalP-5.0/), NLStradamus (http://www.moseslab.csb.utoronto.ca/NLStradamus/), and PredGPI server (http://gpcr.biocomp.unibo.it/predgpi/pred.htm) analysis, respectively, which indicated that TA05575 was secreted into the host cell cytosol and nucleus. In addition, the target protein did not have putative transmembrane domains after using TMHMM server (http://www.cbs.dtu.dk/services/TMHMM/) analysis. While TA05575 did not have FAINT domain based on the SMART and Pfam identification, which was the typical regions found in SVSP multigene family of *T. annulata*. However, the ORF of reference gene TA05575 was 1,401 bp, encoding 316 aa. The similarity of nucleotide and amino acid sequences of TA05575 obtained in this study and reference gene fragment was 96.1 and 92.4%, respectively.

### Analysis for Autoactivation and Toxicity of Bait Plasmid

**Figure 4:**
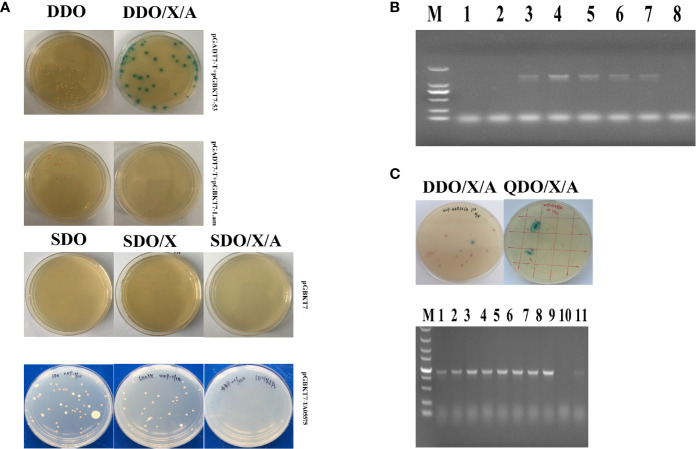
Screen of prey proteins interact with TA05575 by Y2H system. **(A)** Identification for autoactivation and toxicity of pGBKT7-TA05575 in Y2H Gold cells. **(B)** Confirmation for the transformation efficiency of pGBKT7-TA05575 recombinant bait plasmid. The colonies grown on SDO plates were randomly selected and performed to colony PCR using specific primers for TA05575. M: DL 2000 DNA marker; 1-8: the colonies picked from the SDO plate. **(C)** Screening and identification of the possible interacting proteins with TA05575. The co-transformants of pGBKT7-TA05575 and cDNA library of bovine B cells were cultured on DDO/X/A and then grown onto QDO/X/A plates. The co-transformants of pGADT7-T and pGBKT7-Lam plasmids, and pGADT7-T and pGBKT7-53 plasmids grown on DDO an DDO/X/A plates were served as negative and positive controls, respectively.

The recombinant TA05575-pGBKT7 bait plasmid was successfully obtained after sequencing identification. Then, the characteristics of autoactivation and toxicity of TA05575-pGBKT7 bait plasmid was identified before Y2H screening experiment. As shown in **Figure 4A**, the size of colonies of the bait plasmid cultured on the SDO and SDO/X plates was almost same as the colonies with the control plasmid-pGBKT7 on the same plates. The results demonstrated that TA05575 bait was not toxic in present study. In addition, for bait plasmid, the color of colonies grown on the SDO and SDO/X plates was white and pale blue (**Figure 4A**), which indicated that autoactivation feature of the bait plasmid was not exist in Y2H system. And the results of the colony PCR showed that the transformation efficiency of TA05575 bait plasmid was 71.5% (**Figure 4B**).

### Y2H Screening and Identification for Potential Prey Proteins

The potential proteins interact with TA05575 was screened using Y2H system. In this study, the blue colonies for the co-transformants of TA05575 and prey plasmid grown on DDO/X/A plates were picked and cultured on QDO/X/A plates to identify the putative interacting proteins with bait protein (**Figure 4C**). The screened prey plasmids were further confirmed by colony PCR and prey plasmids rescue. The nucleotide sequences of potential plasmids were determined using the BLAST Tool in NCBI. With the BLAST results, the screened prey plasmid had 99.68% similarity with Bos taurus RNA binding motif protein, X-linked 2-like (LOC534630) (RBMX2-like, Accession No: NM_001206548.1). The fragments we screened contained the CDS region from 412 to 1,041 nucleotides of RBMX2-like mRNA, encoding 210 amino acids. And RBMX2-like protein contained a RNA recognition motif (RRM) at the residues at 36 to 114 aa, the biological process of this protein was involved in mRNA splicing *via* spliceosome based on bioinformatic analysis of UniProt and SMART servers. The results of STRING (https://version11.string-db.org/cgi/input.pl?sessionId=1zlnqDY4pkmx&input_page_show_search=on) server indicated that predicted functional partners with RBMX2-like protein included Bos taurus BUD13 homolog, Bos taurus Smad nuclear interacting protein 1 (SNIP1), Splicing factor 3b, subunit 1 and subunit 2 even subunit 3 and Bos taurus PHD finger protein 5A, and so on.

### Expression and Subcellular Distribution of TA05575 and Its Putative Interact Protein

**Figure 5:**
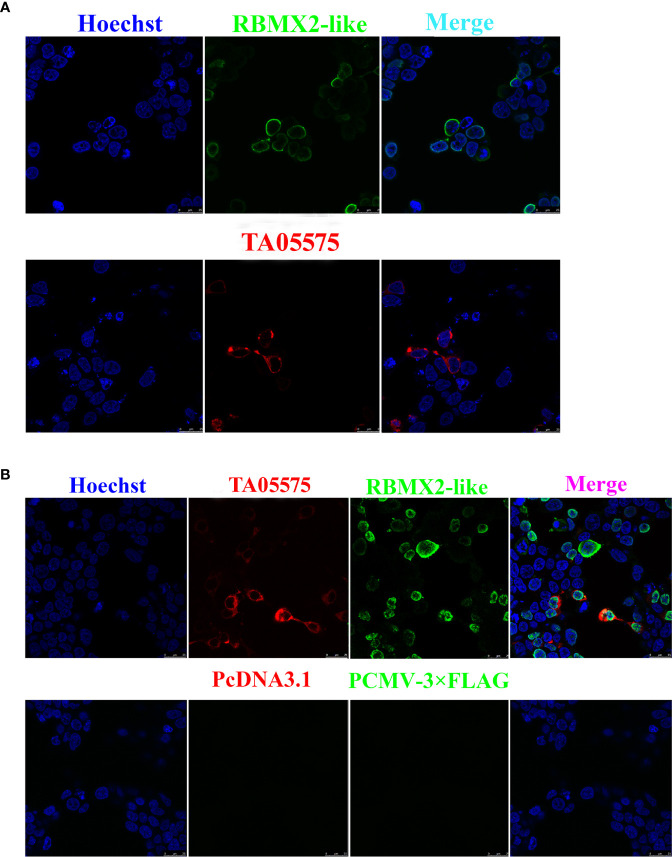
Expression and subcellular distribution analysis of TA05575and its potential interacting protein in HEK293T cells. Confocal microscopy analysis of HEK293T cells expressing TA05575 (4 µg) and its possible prey protein-RBMX2-like (4 µg) **(A)**, and (TA05575 (2 µg)-RBMX2-like (2 µg) **(B)**, respectively. Scale bar = 25 μm.

The recombinant plasmids of Myc-tagged TA05575 and its potential interacting protein (Flag-tagged RBMX2-like) were successfully constructed. The findings of immunofluorescence assay showed that Myc-tagged TA05575 and Flag-tagged RBMX2-like were both successfully expressed when they were individually or pairwise transfected in HEK293T cells, and the subcellular distribution of these proteins was mainly visualized in the cytoplasm of cells based on the confocal microscopy experiment (**Figures 5A, B**). More importantly, we found that Myc-tagged TA05575 and Flag-tagged RBMX2-like protein were colocalized in the cytoplasm of HEK293T cells, which suggested that TA05575 could interplay with RBMX2-like protein (**Figure 5B**).

### Identification of the Interaction of TA05575 With RBMX2-Like by Co-IP

**Figure 6:**
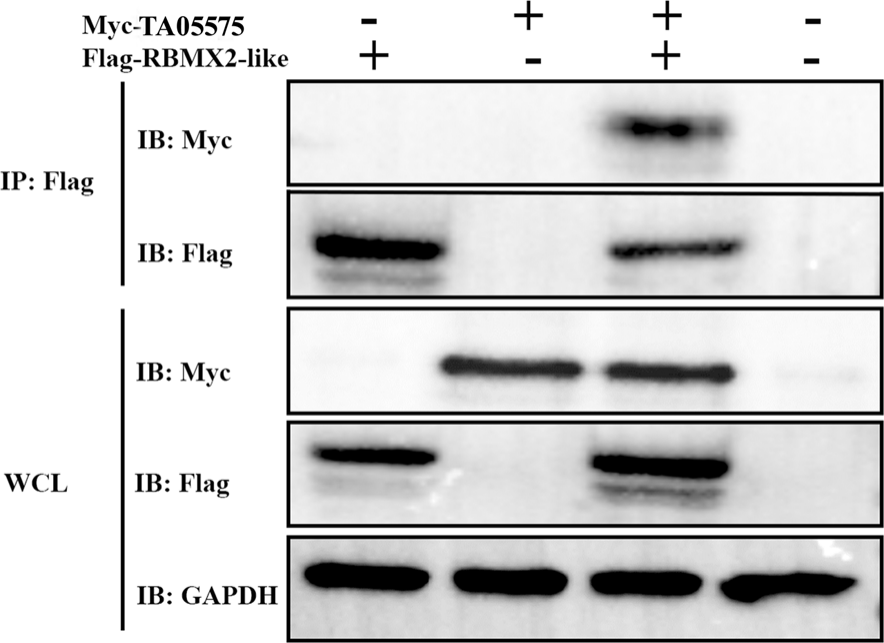
TA05575 interacts with RBMX2-like in HEK293T cells. Co-IP and immunoblotting (IB) detection of the cells expressing TA05575 (10 µg) and its potential pair RBMX2-lik**e** (10 µg). Whole HEK293T cells (WCL) represents IB analysis of the cell lysates for transfection without IP.

After determination of expression and subcellular distribution of TA05575 and its potential interacting partner, we confirmed the interactions between TA05575 and RBMX2-like in the HEK293T cells using the Co-IP assay (**Figure 6**). The results of our study demonstrated that TA05575 interacted with RBMX2-like protein, which further supported the findings of confocal microscopy (**Figure 5B**).

### TA05575 Binds Directly to RBMX2-Like Protein in Cell Context

**Figure 7:**
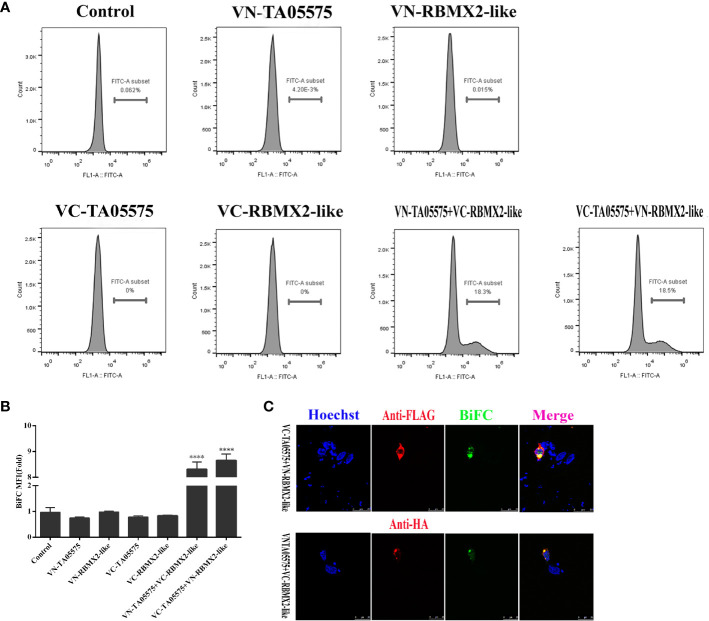
TA05575 directly binds to RBMX2-like in a cell context. TA05575-VN (4 μg), SVSP449-VC (4 μg), RBMX2-like-VN (4 μg), RBMX2-like-VC (4 μg), TA05575-VN (2 μg)-RBMX2-like-VC (2 μg), and TA05575-VC (2 μg)-RBMX2-like-VN (2 μg) were expressed individually or pairwise in HEK293T cells. The MFI of the green-fluorescent signals for BiFC assay was determined using flow cytometry, and the value of MFI is relative to fluorescent signal of un-transfected HEK293T cells, which were served as the control **(A**, **B)**. HEK293T cells were co-transfected with TA05575-VN (2 μg)-RBMX2-like-VC (2 μg), TA05575-VC (2 μg)-RBMX2-like-VN (2 μg) pairs. The subcellular colocalization of the red of interacting pairs and green-fluorescent signals for BiFC were visualized by confocal microscopy **(C)**. Scale bar =25 μ m. ****p < 0.0001.

To further determine the interaction of TA05575 with RBMX2-like protein in cellular context, BiFC assay was carried out. In the present study, the results showed that no fluorescent signals were detected by flow cytometry method when the plasmids of TA05575-VN, TA05575-VC, RBMX2-like-VN, RBMX2-like-VC were individually transfected in HEK293T cells (**Figures 7A, B**). However, strong fluorescent signals were observed when TA05575-VN and its complementary RBMX2-like-VC or TA05575-VC and RBMX2-like-VN pairs were co-transfected into HEK293T cells (**Figures 7A, B**).

The specificity and subcellular colocation of TA05575-RBMX2-like pair was further determined by visualizing the green BiFC fluorescent signals with confocal microscopy. The strong BiFC green fluorescent signals were observed from co-transfected TA05575-VN/RBMX2-like-VC or TA05575-VC/RBMX2-like-VN pairs in cells (**Figure 7C**), which was consistent with the findings of flow cytometry. Moreover, the green signals of TA05575-RBMX2-like interacting pair colocalized with the red fluorescent signals of RBMX2-like demonstrated their perinuclear subcellular localization in HEK293T cells (**Figure 7C**). These above findings demonstrated that TA05575 of *T. annulata* interplayed with bovine RBMX2-like protein in the intracellular compartments of cell contexts.

## Discussion

n the present study, a member of SVSP family, TA05575 was used as a target molecule to investigate its role in host-parasite interactions. Previous findings have analyzed the expression profiles of some SVSP molecules in *T. parva* (Schmuckli et al., 2009) and some results using bioinformatics in *T. annulata* (Weir et al., 2010), however, the expression characteristics of this multigene family in various life cycle stages of *T. annulata* and *T. annulata*-infected cells was not investigated. The findings of our research firstly determined that TA05575 was mainly expressed in the schizont stage in *T. annulata*, which implied it was closely associated with *T. annulata* pathogenecity and in consistent with the previous speculations. Meanwhile, the subcellular localization of TA05575 was observed in different cell culture passages (F10, F20, F55, F110, and F165) in *T. annualta*-infected cells, the results were similar with the CELLO v.2.5 prediction that TA05575 was mainly distributed in the nucleus and cytoplasm compartments in *T. annulata*-transformed cells, and the regions of subcellular distribution was basically not changed when the cell culture passages varied. The above findings indicated that TA05575 was likely to execute its function by manipulating the host cell molecules.

Herein, to find the possible interacting proteins of host cells with TA05575, Y2H screening system was used in the present study. Finally, The RBMX2-like protein of bovine was identified after sequencing and bioinformatic analysis. To confirm whether the RBMX2-like protein interacts with TA05575, we used Co-IP assay to investigate the interplay between bait and prey proteins in HEK293T cells, and the findings demonstrated that the host cell protein-RBMX-2 like interacted with TA05575, which was further verified the observations of confocal microscopy. To further investigate the interactions of TA05575 and its prey protein, BiFC assay was performed in our study. Both results of confocal microscopy and flow cytometry demonstrated that TA05575-RBMX-2 like pair directly interacted and the subcellular colocalization region is in the cytoplasm of HEK293T cells.

RBMX2-like encodes the protein containing a RRM domain protein of bovine at residues from 56 to 134 aa. The biological process of RBMX2-like maybe involved in mRNA splicing *via* spliceosome. At present, the specific function of RBMX2-like is not clear, however, serval reports have indicated that it could be used as a molecular marker to assess spermatozoa in MA patients (Ahmadi et al., 2015). Someone found RBMX2 was downregulated in chromosome X in TCs, which might play a role in cell immunology (Zhu et al., 2015), and retention and splicing (RES) complex in RBMX2 was associated with splicing in vertebrate development (Fernandez et al., 2018). Therefore, RBMX2 has a potential role as a molecular marker for *T. annulata* infection due to its unique feature. More importantly, we speculate that the potential alternative splicing of TA05575 is by stabilizing RBMX2-like protein of bovine, and this biological process maybe further affect the parasite transformation in host cells.

In summary, we discovered the TA05575 was mainly expressed in schizont stage of *T. annulata*. Moreover, the subcellular distribution of TA05575 was determined in different cell passages of *T. annualta*-infected cells. The above findings will pave path to elucidate the role of TA05575 during *T. annulata* infection in the future study. More importantly, we firstly identified RBMX2-like interacted with TA05575 using Y2H system, Co-IP, and BiFC assays. In brief, the investigations into the interactions of *T. annulata* and host cells could provide key insights into the basic biology underlying cancer-like phenotypes.

## Data Availability Statement

The raw data supporting the conclusions of this article will be made available by the authors, without undue reservation.

## Ethics Statement

The animal study was reviewed and approved by the Animal Ethics Committee of the Lanzhou Veterinary Research Institute, Chinese Academy of Agricultural Sciences.

## Author Contributions

ZL, JLL, and HY conceived and designed the experiments. ZL, QM, and SZ performed the experiments. ZL, JLL, AL, YL, GG, and JXL analyzed the data. ZL drafted the manuscript. All authors contributed to the article and approved the submitted version.

## Funding

This study was supported by the National Key R&D Program of China (2017YFD0500403), National Natural Science Foundation of China (31402189, 31972706), ASTIP, NCBIS (CARS-37), and Central Public-interest Scientific Institution Basal Research Fund (1610312016009), and Jiangsu Co-innovation Center for Prevention and Control of Important Animal Infectious Diseases and Zoonoses, State Key Laboratory of Veterinary Etiological Biology Project.

## Conflict of Interest

The authors declare that the research was conducted in the absence of any commercial or financial relationships that could be construed as a potential conflict of interest.

## Abbreviations

SD/-Trp (SDO), synthetically defined medium without tryptophan; SD/-Trp/X-α-Gal (SDO/X), synthetically defined medium without tryptophan supplemented with X-α-Gal; SD/-Trp/X-α-Gal/AbA, synthetically defined medium without tryptophan supplemented with X-α-Gal; SDO/X/A, synthetically defined medium without tryptophan and leucine supplemented with X-α-Gal and aureobasidin A; DDO, synthetically defined medium without tryptophan and leucine; DDO/X/A, synthetically defined medium without tryptophan and leucine supplemented with X-α-Gal and aureobasidin A; QDO/X/A, synthetically defined medium without tryptophan, leucine, adenine, histidine supplemented with X-α-Gal and aureobasidin A.
